# Pre‐gerotal fat patch—A novel alternative to haemostatic agents during partial nephrectomy

**DOI:** 10.1002/bco2.264

**Published:** 2023-08-16

**Authors:** Brennan Timm, Alice Thomson, Damien Bolton, Michael Pether

**Affiliations:** ^1^ Bunbury Regional Hospital Bunbury Western Australia Australia; ^2^ Austin Health Heidelberg Victoria Australia

**Keywords:** haemostatic bolster, minimally invasive surgery, open surgery, partial nephrectomy, pre‐gerotal fat patch

## Abstract

**Objective:**

This study aimed to determine if using a pre‐gerotal fat patch at open partial nephrectomy (PN) as a haemostatic bolster is a viable alternative to using synthetic haemostatic agents.

**Materials and methods:**

Human Research Ethics Committee approval was obtained for audit of a prospectively kept database from July 2012 to July 2021, which followed outcomes of patients who received a low‐tension pre‐gerotal fat patch renorrhaphy at open PN. Patient demographics, intraoperative measures, histological outcomes and post‐operative complications were analysed. Using a retroperitoneal approach, the peritoneum was mobilised and a vascularised pedicle of pre‐gerotal fat was rotated in the direction of the kidney. Routine definition of the hilum, clamping of the hilar vessels and dissection of mass followed. After watertight closure and haemostasis, the harvested pre‐gerotal fat patch was placed over the defect and secured using low‐tension renorrhaphy. Two‐layer closure of the abdominal wall with placement of a drain was routine.

**Results:**

A total of 55 patients underwent open PN. Mean age was 60.4 (35–77) years. There were 38 men and 17 women, and 32 right and 23 left PNs. Mean mass size was 31.9 mm (10–95 mm) and collecting system was breached in 36.5% of cases. One patient (1.9%) suffered a Clavien–Dindo IIIb complication requiring return to theatre and transfusion due to a bleed from an intercostal artery. There were no renal bed bleeds, urine leaks or urine fistulas detected. Mean intraoperative blood loss was 355 mL (50–1500 mL) and mean post‐operative creatinine increased by 10.7 μmol/L (51–172 μmol/L). Mean follow up was 40.2 (4–109) months.

**Conclusion:**

Utilisation of an anatomical pre‐gerotal fat patch to provide pressure at the renorrhaphy site during open PN is an effective technique to assist with surgical haemostasis. This simple technique avoids the costs of haemostatic agents, whilst adding minimal operating time to procedures.

## 1 INTRODUCTION

Contemporaneously, most small renal masses (SRMs) are incidentally diagnosed at imaging for unrelated conditions.^1^ Most international guideline statements have adopted renal preservation techniques as standard of care, in order to decrease secondary impacts of later diabetes mellitus or cardiovascular disease on renal function.^2^, ^3^ Partial nephrectomy (PN), by either an open or minimally invasive approach, remains the preferred treatment for T1a–b masses due to its favourable functional and oncological outcomes as compared with radiation, thermoablation or radical nephrectomy (RN).^4^ PN is being expanding to more challenging clinical scenarios, such as for larger lesions and in less favourable positions.^5^, ^6^

**FIGURE 1:**
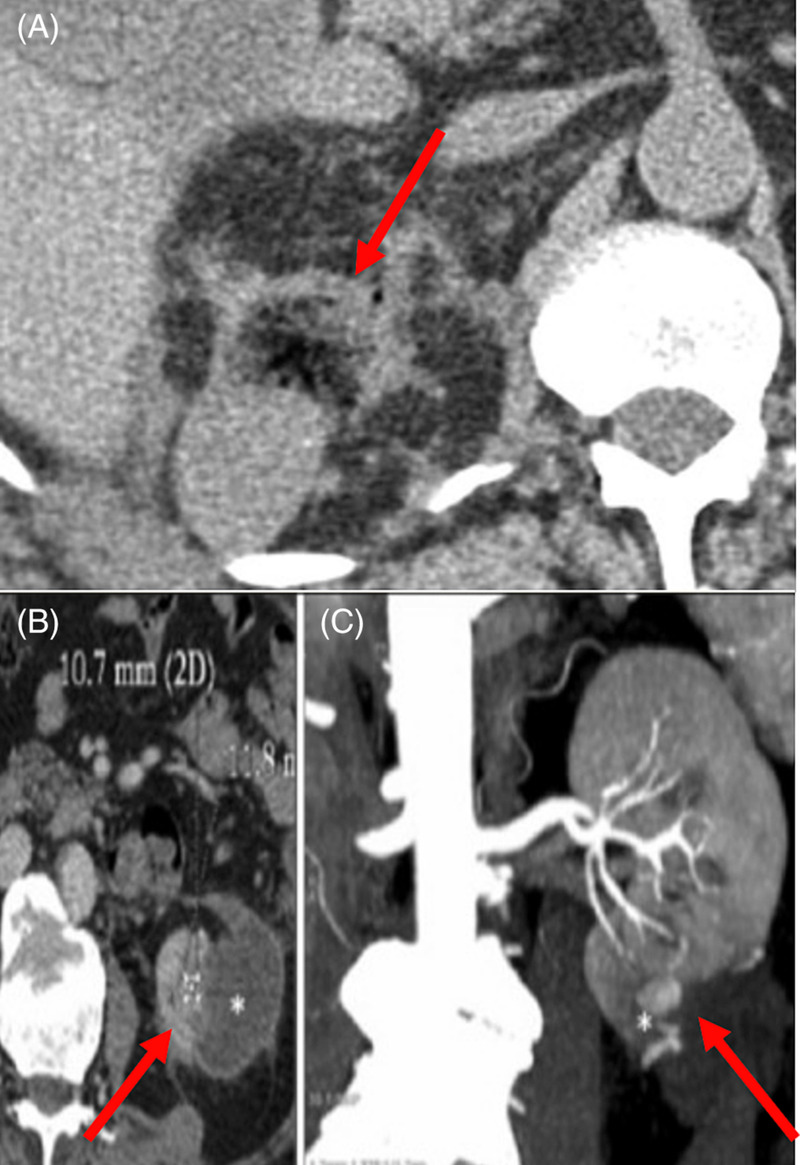
(A–C) Post‐partial nephrectomy surveillance computed tomography scans demonstrating local enhancement and air locules at site of resection (red arrows), when synthetic haemostatic bolsters have been used.

Nonetheless, PN is associated with the potential for adverse outcomes including haemorrhage requiring transfusion, urine leak and fistulas, which have been reported in up to 13%, 17% and 17% of patients, respectively.^2^, ^3^, ^7^ Additionally, issues with post‐operative radiological assessment of possible tumour recurrence post‐PN may ensue due to synthetic haemostatic bolster granulomas limiting accurate interpretation of underlying soft tissue (Figure 1).^8^, ^9^, ^10^ When such issues in imaging arise, patients may require additional, potentially avoidable and costly investigations or treatment.^11^

**FIGURE 2:**
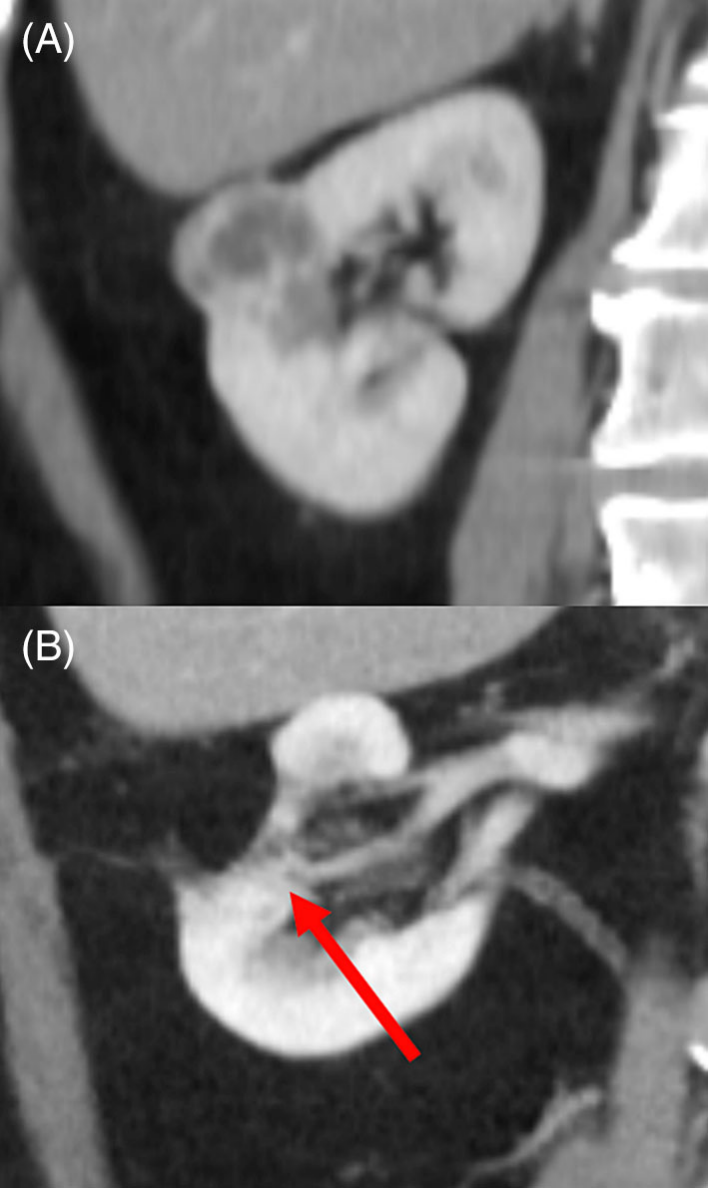
Computed tomography images of (A) pre‐ and (B) post‐partial nephrectomy, when a pre‐gerotal fat patch has been used as a haemostatic bolster.

In an effort to reduce potential morbidity, improve post‐operative imaging interpretation (Figure 2) and reduce intraoperative costs, we audited clinical outcomes from consecutive open PN (OPN) cases where pre‐gerotal fat on a pedicle and low‐tension renorrhaphy was routinely used as an alternative to other renorrhaphy closure techniques. We compared outcomes including cost, operative time and morbidity with published works on PN using synthetic haemostatic agents and high tension renorrhaphy to evaluate relative outcome measures.

## 2 MATERIALS AND METHODS

Human Research Ethics Committee approval was obtained from the Western Australia Country Health Service Ethics Committee (LNRP 2021.04) for an audit of a prospectively kept database from July 2012 to July 2021, which followed outcomes of patients who received a low‐tension pre‐gerotal fat patch renorrhaphy at OPN. Patient demographics, blood loss, operative time, length of stay (LOS), histological outcomes and post‐operative complications were recorded and analysed.

**FIGURE 3:**
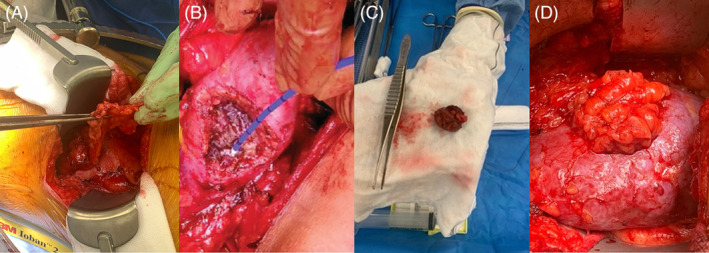
(A) Harvested pre‐gerotal fat patch. (B) Defect in renal parenchyma following excision of lesion. (C) Excised lesion. (D) Tension‐free renorrhaphy with pre‐gerotal fat patch over defect.

The surgical technique utilised for all patients was a tip of 12th rib flank incision and retroperitoneal approach. Following mobilisation of peritoneum antero‐medially, the pre‐gerotal fat was mobilised by right angle dissection to a size adequate to drape over the site of planned resection, and developing a vascularised pedicle graft in the direction of the defect (Figure 3A). Definition of the renal mass and hilum, application of a cross‐clamp (either of the main artery or of a branch thereof dependent on mass locality) and dissection of the mass utilising monopolar diathermy were routine (Figure 3B,C). Vessels on the resection surface were oversewn using 2‐0 G123 chromic gut and collecting system defects were closed with 5‐0 PDS. After watertight closure and haemostasis were achieved and assessed post removal of the arterial cross‐clamp, the previously harvested pre‐gerotal fat patch is untucked and placed gently over the defect. Low‐tension renorrhaphy to secure the fat pad was completed with three or four (dependent on size of defect) 2‐0 G123 chromic gut to gently oppose fat onto resection surface (Figure 3D). A two‐layer muscular closure with placement of a drain was routine.

## 3 RESULTS

A total of 55 consecutive patients underwent OPN. Mean age was 60.4 (35–77) years. There were 38 men and 17 women, and 32 right and 23 left PNs. Preoperative renal biopsy was performed in 21.2% with 100% concordance of biopsy and final histology.

Operative time was 167.7 (90–240) min, cross‐clamp time was 12.2 (4–25) min, and average LOS was 5 (3–10) days.

The collecting system was breached in 36.5% of cases. Mean intraoperative blood loss was 355 mL (50–1500 mL).

Mean mass size was 31.9 (10–95 mm). Histology was 57.7% clear cell, 19.2% papillary, 11.5% oncocytoma, 3.8% chromophobe and 3.8% angiomyolipoma (AML) (Table 1). Four (7.7%) patients had a resection margin in contact with the cancer.

**TABLE 1:** Patient demographics.

		Mean (range)
Age (years)		60.4 (35–77)
M:F		38:17
R:L		32:23
Preop biopsy		21.2%
Final histopathology	Clear cell	31
	Chromophobe	2
	Oncocytoma	7
	Papillary RCC	11
	Angiomyolipoma	3
Size of lesion (mm)		31.9 (10–95)
Operation time (min)		167.7 (90–240)
Cross‐clamp time (min)		12.2 (4–25)
EBL (mL)		355 (50–1500)
Collecting system breached		36.5%
LOS (days)		5 (3–10)
Margin in contact		7.7%
Change in creatinine (μmol/L)		10.7 ± 3.2
Recurrence		3.8%
Follow up (months)		40.2 (4–109)
Complications		1.9%

Abbreviations: EBL, estimated blood loss; LOS, length of stay; RCC, renal cell carcinoma.

One patient (1.9%) suffered a Clavien–Dindo IIIb complication requiring return to theatre and transfusion due to a bleed from an intercostal artery. There were no occasions of significant renal bed haemorrhage, urine leak or urine fistula detected. Mean post‐operative creatinine increased by 10.7 (51–172 μmol/L) (Table 2).

**TABLE 2:** Risk of complications following open partial nephrectomy: Pre‐gerotal fat patch audit compared with open partial nephrectomy review.

	Pre‐gerotal patch audit	TAU 2020 OPN review^8^
	Risk %	
Major complication	1.9	2–9.0
**Intraoperative**		
Bleed requiring transfusion	0	1.2–5.3
Damage to other organ	0	0.4–2.6
**Early post‐operative**		
AKI	3.8	2.4–5.4
Urine leak	0	1.4–17
Renal bed bleed	0	1.5–8
Bleed requiring transfusion	1.9	1.5–13
**Late post‐operative**		
Urinary fistula	0	1.4–17.4
AV fistula	0	<1

Abbreviations: AKI, acute kidney injury; AV, arteriovenous; OPN, open partial nephrectomy; TAU, *Translational Andrology and Urology*.

Average creatinine was 84 μmol/L, rising to 95 μmol/L post‐operatively, a difference of 10.7 μmol/L (95% confidence interval [CI] 3.2 μmol/L).

Mean follow up was 40.2 (4–109) months. At the time of writing, radiologic detected recurrence had ensued in two patients: one in contact with a positive margin and another in adjacent para‐aortic lymph nodes.

## 4 DISCUSSION

PN relies on an understanding of renal vasculature and segmental arterial flow, permitting partial or complete occlusion of renal parenchymal blood flow. Approaches may be varied to minimise nephron ischaemia and its potential to decrease renal function.^12^ In cases of complex renal masses, significant pre‐existing renal impairment, repeat PN on the same kidney, solitary kidneys, multiple or bilateral tumours or a lack of minimally invasive PN (MIPN) expertise, OPN remains an accepted approach.^13^ Whilst MIPN and OPN continue to have equivalent oncological and functional outcomes, both have been shown to have certain advantages.^4^, ^14^ MIPN lacks the potential for intraoperative cooling and has been shown to have longer ischaemic times than OPN.^15^ MIPN provides for better surgeon ergonomics and shorter patient convalescence secondary to more favourable incision placement.^14^, ^16^

MIPN is being used on increasingly difficult clinical cases, particularly with greater access and operative comfort with robotic platforms. Whilst PN was typically reserved for small lesions, lesions larger than 7 cm are now being considered for PN with good oncological outcomes, as well as endophytic lesions.^5^, ^6^ Additionally, surgeons are attempting more cases off‐clamp, decreasing the effect of ischaemia on renal function, without significant increase in bleeding for appropriately selected patients.^17^, ^18^ Having a readily available autologous haemostat may help improve outcomes in these cases.

Following the decision that MIPN or OPN should be offered as treatment for an SRM, the operative principles are similar. The patient is positioned in the lateral decubitus position, with the affected kidney superiorly. The bed is flexed maximally to increase intra‐abdominal operative space on that side, taking care to not cause any pressure sites.^7^ Entry into the abdomen is achieved through an open retroperitoneal flank incision with use of a retractor or transperitoneal/retroperitoneal laparoscopic ports.^19^ The kidney, hilum and mass are localised, and the vessels are defined.^12^ Overlying adipose is removed from the exophytic margin and intraoperative ultrasound may be used to define deep margins.^20^ Once the resection approach is planned, the vessels are occluded, the mass resected and the defect closed with absorbable suture material.

The specific technique of repair of the renorrhaphy is the variable that is audited in this study. Our low‐tension fat patch renorrhaphy as described above is not technically challenging, takes 3–5 min and prevents ‘cheese wiring’ of the kidney associated with higher tension renorrhaphy techniques.

Complications at PN are not uncommon, with major complication rates reported to range from 2% to 9%.^7^ This series demonstrated no requirement for intraoperative transfusion and no damage to other organs, with those specific outcomes reported in up to 5% and 2.5%, respectively, in other series.^14^, ^21^ In the early post‐operative period, 3.8% of our patients experienced an acute kidney injury, comparable with the 2.4%–5.4% noted in the literature. Significant haemorrhage from any cause requiring a transfusion is seen in 1.5%–8% of patients undergoing PN, whilst in this series, we experienced one such complication.

Early post‐operative outcomes in this series were better than might have been expected, with no patients experiencing a urine leak or a renal bed haemorrhage, which have been reported in up to 17% and 8% of patients, respectively, in other series.^19^, ^21^, ^22^ There were no instances of urinary or arteriovenous fistulas within a minimum of 4‐month follow‐up period. These complications have previously been reported in up to 17% of patients.^22^, ^23^

There was a drop of on average of 10.7 μmol/L (95% CI 3.2 μmol/L) in estimated Glomerular Filtration Rate (eGFR), which is slightly greater than reported in the literature.^8^ Performing the procedure off‐clamp has been demonstrated to maintain preoperative renal function compared with on‐clamp procedures.

The technique described for use of an overlay autogenous tissue (OAT) patch has been used in association with other organs for the management of uncontrollable bleeding, where a fascial patch or omentum on a pedicle is placed over the bleeding surface.^24^, ^25^ The consequent improved haemostasis is postulated to be secondary to a combination of the venturi effect the graft places upon the damaged vessel and by creating a frame of fibrin and platelet deposition. An additional pathway demonstrated in animal and in vitro models may be the activation of adipose stem cells and undifferentiated mesenchymal stem cells to stimulate repair processes, whilst also recruiting progenitor cells.^26^, ^27^ These progenitor cells can differentiate into any required tissue type whilst suppressing immune reactions.

Urologists have traditionally avoided the routine incorporation of adipose into surgical defect repair, and in particular, they have preferred to use synthetic haemostatic bolsters for management of renorrhaphy. General surgeons routinely utilise omentum to close duodenal ulcers, oesophageal perforations, and thoracic and biliary duct leaks, as well as to cover hepatic or splenic injuries.^28^ Plastic surgeons regularly use autologous fat grafts, and free fat grafts are regularly applied in controlling cerebral spinous fluid leaks by neurosurgeons.^29^, ^30^

Synthetic haemostatic bolsters are associated with specific issues pertinent to the oncologic basis of PN. Interpretation of post‐operative imaging to exclude tumour recurrence may be compromised by their use. Potential exists for these prostheses to be mistaken for early tumour recurrence, potentially necessitating repeat imaging or costly intervention (Figure 1). A fat patch bolster minimises any risk of imaging misinterpretation post‐operatively (Figure 2). Rare complications include anaphylaxis to animal gelatin containing components (Surgiflo/Floseal), bowel obstruction secondary to regenerated cellulose sheets (Surgicel/TachoSil) and migration of haem‐o‐lok clips into the urinary tract resulting in stone formation. Multiple instances of such outcomes have been documented, and it is likely that the incidence is higher than reported.^8^, ^9^, ^31^, ^32^, ^33^

Costs associated with PN are significantly higher as compared with minimally invasive RN or thermoablation techniques.^34^ This is often secondary to increased LOS in OPN, cost of disposables in robotic‐assisted PN (RAPN) and the use of endoscopic surgical supplies and haemostatic agents in MIPN. Utilising a fat patch limits or removes the need for synthetic haemostatic agents. In MIPN, it would not be uncommon to use one to two 5 × 7.5‐cm sheets of Surgicel® (Johnson and Johnson, NJ, USA), one to two cartridges of 5‐mL Floseal® (Baxter Healthcare, IL, USA) or 4‐mL Tisseel® (Baxter Healthcare) and up to three or four packets of Hem‐o‐Lok® clips (Teleflex®, NC, USA), costing $1000 to ~$1900AUD in total (Table 3). Given the rising costs of healthcare and the need for fiscally responsible choices, using a fat patch may avoid these costs without compromising haemostatic function.

**TABLE 3:** Surgical haemostatic agents costs, January 2021—Australian Government Department of Health prostheses list.

Surgical haemostatic agents	Prostheses list	Average cost for MIPN
Floseal endoscopic applicator	$138	$138
Floseal 5 mL	$632	$632 × 1–2
Surgiflo endoscopic applicator	$138	
Surgiflo 5 mL	$407	
Surgicel SNoW 2.5 × 5	$34	
Surgicel SNoW 5 × 10	$69	$69 × 1–2
Surgicel Fibrillar 2.5 × 5	$34	
Surgicel Fibrillar 5 × 10	$69	
Surgicel powder	$136	
Tisseel 2 mL	$327	
Tisseel 4 mL	$602	
TachoSil 4.8 × 4.8	$208	
Haem‐o‐lok clip cartridge (6 clips)	$62	$62 × 4–6
Total estimated cost		$1087–1912

Abbreviation: MIPN, minimally invasive partial nephrectomy.

Use of an anatomical pre‐gerotal fat patch during PN may not be appropriate if the patient has a low volume of pre‐gerotal fat available or is not suitable to be mobilised and left in situ. In these situations, the operating surgeon must be familiar with alternative haemostatic agents.

## 5 CONCLUSION

Utilisation of an anatomical pre‐gerotal fat patch at OPN is an effective haemostatic technique, which may also reduce the potential for urine leak. It takes only minutes to define and apply, can aid in reducing post‐operative imaging surveillance confusion and concern and reduces reliance upon expensive intraoperative haemostatic agents. Potential exists for a laparoscopic version of this technique to be described. Pre‐gerotal fat patch application is a potentially advantageous technique for urologists undertaking PN and we advocate for its use.

## AUTHOR CONTRIBUTIONS

BT and MP devised the project. BT performed data collection and analysis, and wrote the initial manuscript. AT and DB contributed to and edited the manuscript. All authors were responsible for the final manuscript. MP supervised the project.

## DISCLOSURE OF INTEREST

The authors have no conflict of interest to declare.
